# Draft genome sequence of *Enterobacter ludwigii* strain Cas398 isolated from rhizosphere soil of tobacco in China

**DOI:** 10.1128/mra.00400-26

**Published:** 2026-06-15

**Authors:** Youai Zhang, Qianping Yan, Weimin Wang, Qing Wang, Junni Qi, Feifei Zheng, Haohui Tong, Baoxiang Pan, Yanhui Lu, Xiaoqiang Wang

**Affiliations:** 1 Zhejiang Tobacco Industrial Co., Ltd., Hangzhou, China; 2 Key Laboratory of Tobacco Pest Monitoring and Integrated Management, Tobacco Research Institute of Chinese Academy of Agricultural Sciences, Qingdao, China; 3 College of Agriculture, Tarim University 12483, Alar, China; 4 Guangxi Zhuang Autonomous Region Tobacco Company of CNTC, Nanning, China; University of Maryland School of Medicine, Baltimore, Maryland, USA

**Keywords:** *Enterobacter ludwigii​*, rhizosphere soil, draft genome

## Abstract

We report here the draft genome sequence of *Enterobacter ludwigii* Cas398, a bacterial strain derived from the rhizosphere soil of tobacco in China. The genome is 4,626,841 bp in size, with a G + C content of 54.98%, and encodes 4,321 protein-coding genes.

## ANNOUNCEMENT

*Enterobacter ludwigii* is a gram-negative bacterium belonging to the class Gammaproteobacteria (1). While some *Enterobacter* species act as opportunistic pathogens, others contribute beneficially to soil ecosystems, including nutrient cycling, plant growth promotion, and biocontrol (2, 3). To elucidate the genomic underpinnings of its ecological role, we isolated and sequenced the genome of strain Cas398. Rhizosphere soil was collected from tobacco fields in Enshi (31°14′09″ N, 121°28′50″ E), Hubei Province, China, on 14 September 2023, according to methods described previously (4). After suspending soil in sterile water, serial dilutions were plated onto nutrient agar (NA; peptone, 10.0 g; meat extract, 5.0 g; sodium chloride, 5.0 g; agar, 15.0 g; pH 7.0, per 1 L of medium) and incubated at 28°C for 3 days. Single colonies were picked and subcultured three times on fresh NA medium, yielding strain Cas398. The 16S ribosomal RNA (rRNA) gene of Cas398 was amplified using primers 27F/1492R (5) and sequenced using Sanger sequencing. The spliced sequence (accession number PZ179853) was searched using BLAST on EzBioCloud (https://www.ezbiocloud.net/), showing 99.79% identity with *E. ludwigii* EN-119^T^ (JTLO01000001).

For genomic DNA extraction, a single colony was picked and inoculated into 100 mL of fresh nutrient broth medium at 28°C until stationary phase with shaking. The SDS-based extraction method was used to obtain high-molecular-weight DNA (6), the purity and concentration of which were evaluated by agarose gel electrophoresis and Qubit fluorometry. Sequencing libraries with an average insert size of 270 bp were prepared with NEBNext Ultra DNA Library Prep Kits (New England Biolabs, USA) and sequenced on an Illumina NovaSeq X Plus platform (Illumina, San Diego, CA, USA), generating 7,159,977 paired-end reads (150 bp each) (2.49 Gb). Quality filtering was performed with fastp (7) to remove reads for which more than 40% of bases had a quality score < 15. *De novo* assembly was performed using SPAdes v3.7 (8), and contigs < 1,000 bp were excluded. Annotation was performed with the National Center for Biotechnology Information Prokaryotic Genome Annotation Pipeline (9) and Prokka (10). Unless otherwise noted, all software applications were run with default parameters.

The assembled genome of strain Cas398 comprises 15 contigs with a total length of 4,626,841 bp, with N50 of 2,450,641, G + C content of 54.98%, and an estimated 569× coverage. The genome completeness and contamination were estimated to be 99.97% and 0.34%, respectively, using CheckM2 (11). The genome was annotated with 4,321 protein-coding genes. In addition, transfer RNA (tRNA) genes were predicted with tRNAscan-SE v1.3.1 (12), and rRNA operons were identified using RNAmmer v1.2 (13). The genome contains 68 tRNAs, 3 5S rRNA, 1 16S rRNA, and 1 23S rRNA. Average nucleotide identity analysis (14) indicated that Cas398 was closely related to *E. ludwigii* EN-119^T^ (CP017279.1) with an OrthoANIu value of 99.02%.

## Data Availability

This Whole Genome Shotgun project has been deposited at GenBank under accession number JBWJHC000000000. The version described in this paper is version JBWJHC000000000.1. The raw sequencing reads generated from Illumina platforms have been submitted to SRA, and the corresponding accession number is SRR37736899.
